# *In planta *gene expression analysis of *Xanthomonas oryzae *pathovar *oryzae*, African strain MAI1

**DOI:** 10.1186/1471-2180-10-170

**Published:** 2010-06-11

**Authors:** Mauricio Soto-Suárez, Diana Bernal, Carolina González, Boris Szurek, Romain Guyot, Joe Tohme, Valérie Verdier

**Affiliations:** 1 UMR 5096 IRD-CNRS-Université de Perpignan, Laboratoire Génome et Développement des Plantes, Institut de Recherche pour le Développement, 911 Avenue Agropolis BP 64501, 34394 Montpellier Cedex 5, France; 2 Biotechnology Research Unit, Centro Internacional de Agricultura Tropical (CIAT), A.A. 6713, Cali, Colombia; 3 Laboratorio de Microbiología Molecular, Centro de Biotecnología y Bioindustria, Corporación Colombiana de Investigación Agropecuaria (Corpoica), Km 14 via a Mosquera, Cundinamarca, Colombia

## Abstract

**Background:**

Bacterial leaf blight causes significant yield losses in rice crops throughout Asia and Africa. Although both the Asian and African strains of the pathogen, *Xanthomonas oryzae *pv. *oryzae *(*Xoo*), induce similar symptoms, they are nevertheless genetically different, with the African strains being more closely related to the Asian *X. oryzae *pv. *oryzicola *(*Xoc*).

**Results:**

Changes in gene expression of the African *Xoo *strain MAI1 in the susceptible rice cultivar Nipponbare were profiled, using an SSH *Xoo *DNA microarray. Microarray hybridization was performed comparing bacteria recovered from plant tissues at 1, 3, and 6 days after inoculation (dai) with bacteria grown *in vitro*. A total of 710 bacterial genes were found to be differentially expressed, with 407 up-regulated and 303 down-regulated. Expression profiling indicated that less than 20% of the 710 bacterial transcripts were induced in the first 24 h after inoculation, whereas 63% were differentially expressed at 6 dai. The 710 differentially expressed genes were one-end sequenced. 535 sequences were obtained from which 147 non-redundant sequences were identified. Differentially expressed genes were related to metabolism, secretion and transport, pathogen adherence to plant tissues, plant cell-wall degradation, IS elements, and virulence. In addition, various other genes encoding proteins with unknown function or showing no similarity to other proteins were also induced. The *Xoo *MAI1 non-redundant set of sequences was compared against several *X. oryzae *genomes, revealing a specific group of genes that was present only in MAI1. Numerous IS elements were also found to be differentially expressed. Quantitative real-time PCR confirmed 86% of the identified profile on a set of 14 genes selected according to the microarray analysis.

**Conclusions:**

This is the first report to compare the expression of *Xoo *genes *in planta *across different time points during infection. This work shows that as-yet-unidentified and potentially new virulence factors are appearing in an emerging African pathogen. It also confirms that African *Xoo *strains do differ from their Asian counterparts, even at the transcriptional level.

## Background

*Xanthomonas oryzae *pv. *oryzae (Xoo) *is the causal agent of bacterial leaf blight in rice. Bacterial cells on leaf surfaces enters the rice leaf by either swimming passively through the fluid oozing from hydathodes in the morning and spreading systemically in the plant through the xylem, or it enters directly into the xylem through wounds [1]. In Asia, this disease is the most economically important within the irrigated environment. It appeared in Africa in the 1980s, and has since been growing in importance [2]. The use of varietal resistance is a highly efficient way of controlling the disease in Asia, but, in Africa, adequate control methods and deployment of resistant varieties are still lacking. Among the prerequisites for finding adequate control strategies are an understanding of the biology of the host-pathogen interaction and the characterization of those genes involved in pathogenicity.

Numerous studies [1] have been carried out on the interaction between both host (rice) and pathogen (Asian *Xoo *strains). In Asia, *Xoo *shows important variations, as revealed by virulence and DNA fingerprinting analyses [3-5]. A race is a group of strains sharing common phenotype of virulence to a set of host cultivars. In the case of *Xoo *near isogenic lines (IRBB lines) are being used and more than 30 *Xoo *races have been reported in Asia so far. New ones are emerging that overcome deployed resistance [6]. Identification of the genes used by the bacteria to colonize plants may give new insights into the plant defence pathways that are vulnerable to pathogen attack and provide better understanding of the processes in both bacterial pathogenesis and plant immunity.

Microarray technology has been widely used to explore transcriptional profiles in plant pathogenic bacteria such as *Pseudomonas syringae*, *Ralstonia solanacearum*, *Xanthomonas axonopodis*, *X. campestris*, and *Xylella fastidiosa *[7-15]. These analyses were conducted to study responses to environmental factors such as heat shock, changes in iron bioavailability or carbon sources [7-9], expression changes related to pathogenesis [10-13], and biofilm formation [13]. Another significant field of microarray analysis is that of genome diversity [14] and horizontal gene transfer events [15], using comparative genome hybridization. One example was the recent development of an *Xanthomonas oryzae *5K oligoarray, with oligos designed according to the sequences of the genomes of Asian strains of *Xoo *and *X. oryzae *pv. *oryzicola *(*Xoc*) [16]. *Xoc *is the causal agent of bacterial leaf streak, a non-vascular counterpart of *Xoo *[1]. *Xoo *and *Xoc *showed differentially expressed genes when grown in enriched versus minimal media [16]. For example, the minimal medium XOM2 induces the *in vitro *expression of the *hrp *genes in *Xoo *but not in *Xoc*, presumably by mimicking the pH and nutrient content in the apoplast [17]. The great potential of microarray technology was also demonstrated by several other studies that used the technique based on whole or partial plant-bacterial genomes [18-20]. Most analyses addressing bacterial gene expression were conducted under *in vitro *conditions.

Strain variations were recently documented in whole-genome analyses of three *Xoo *strains: the Korean *Xoo *strain KACC10331 [21], the Japanese *Xoo *strain MAFF311018 [22], and the Philippine *Xoo *strain PXO99^A ^[23]. A whole-genome sequence is also available for one Asian *Xoc *strain BLS256. Several characteristics differentiate the *Xoo *genome from those of other xanthomonads: a higher abundance of IS elements, and prevalence of TAL effector genes of the *avrBs3*/*pthA *family [1,22]. TAL genes are widespread among *Xanthomonas *spp., but this family of effectors has expanded specifically in the genomes of Asian *X. oryzae *pathovars. Recent studies identified African *Xoo *strains as a significantly different genetic group that appears more closely related to the Asian *Xoc *than to Asian *Xoo *[24]. In contrast to Asian *Xoo *strains, African *Xoo *strains show a reduced number of both TAL genes and IS elements in their genomes [24]. African *Xoo *strains induce a non-host hypersensitive response (HR) in tobacco leaves suggesting that these strains display one or several specific non-host HR elicitors, such as type III effectors or harpins. Finally, three new races have been determined among the African strains [24]. However, except for the role of one TAL effector, almost nothing is known about the specific genetic determinants of pathogenicity in *Xoo *African strains (Yu Y., Szurek B., Mathieu T, Feng X., Verdier V. 2009, unpublished data). Much remains to be learned about the genes involved in the pathogenicity and virulence of this African pathogen. Identification of such genes can improve understanding of how *Xoo *causes disease.

Efficient methods for recovering bacterial cells directly from plant tissues permit analyses of *in vivo *expression in plant-pathogen interactions [25,26]. Conducting gene expression analyses of bacterial pathogens *in planta *may improve the understanding of the mechanisms underlying plant-pathogen interactions and may help in the early detection of genes involved in pathogenicity [25,27]. Because whole genome is not yet available for African *Xoo *strains, we used SSH libraries of *Xoo *strain MAI1 [28] that were then spotted onto a microarray and used to analyse *in planta *gene expression at different time points during infection. Combining the SSH method, *in vivo *analysis, and microarrays to study the *Xoo *MAI1-rice interaction offers considerable advantages, particularly as *in vitro *approaches are frequently limited in their ability to mimic all aspects of the *in vivo *state. Aditionally, constructing an *Xoo *MAI1 microarray, based on SSH DNA libraries, allows the enrichment of *Xoo *MAI1 sequences. Hence, the likelihood is higher that the microarray will reveal novel genes involved in *Xoo*-rice infection. Although the *Xoo *MAI1 SSH-microarray does not allow analyses of genome-wide gene expression profiles, specific biological questions can be answered more efficiently, for example, identification of virulence determinants in African *Xoo *strains. In contrast with other large-scale approaches to the study of gene expression in the *Xanthomonas *genus [10,16,25,29,30], this is the first report to compare bacterial gene expression *in planta *and at different time points during infection.

## Results and discussion

### Bacterial recovery from plant tissues, and RNA isolation

**Figure 1:**
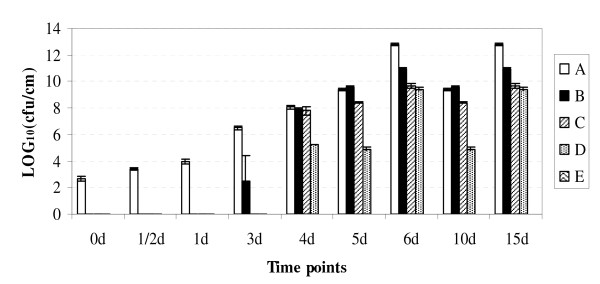
***In planta *quantification of bacteria**. Bacterial growth in 8-week old rice variety Nipponbare, in sections A, B, C, D, and E of the leaf at 0 and 12 h, and 1, 3, 6, 10, and 15 days after inoculation. The experiment was repeated three times with three leaves per time point. Error bars indicate standard errors.

We determined *Xoo *MAI1 multiplication *in planta *at seven time points after infection into five 2-cm leaf sections (A-E, Figure 1). The *Xoo *strain MAI1 multiplied to a population size of almost 10^-4 ^colony-forming units (cfu) in section A within 12 h after inoculation (hai). Thereafter, the population continued increasing until it reached a size of more than 10^-12 ^cfu within 15 days after inoculation (dai; Figure 1). That is, colonization along the leaf was fast. Initially, *Xoo *bacterial cells were concentrated in the first 2 cm behind the inoculation point but, within 3 dai, they were found in section B. By day 6, the bacterium had colonized more than 8 cm, reaching section D. Levels of *Xoo *MAI1 populations increased gradually from sections A to D, reaching 10^-9 ^to 10^-13 ^cfu per section of leaf by 15 dai. By that time, visible lesions were about 10 cm long. We selected three time points (1, 3, and 6 dai) and the first 2-cm lesion to perform bacterial RNA extractions from leaf tissues for subsequent microarray experiments. Possible genomic DNA contamination was tested by PCR, using primers corresponding to the genomic region flanking the *hrpX *(hypersensitive reaction and pathogenesis) gene and purified RNA as PCR template. No DNA contamination was found (data not shown).

### Differentially expressed genes were identified at late stages of infection

The DNA microarray constructed consists of about 4708 randomly selected clones. The quality of PCR amplification was verified for 20% of the amplified genes (1330 clones), with sizes ranging from 600 to 900 bp. The arrays were hybridized with Cy labelled cDNA probes prepared from total RNA from plant-grown bacteria at 1, 3, and 6 dai, or from bacteria cultured in media and re suspended in water.

We used bootstrap analysis with SAM to identify differentially expressed genes. Significance Analysis of Microarrays (SAM) calculates the fold change and significance of differences in expression. The delta-delta Ct values ranged from 1.21 to 2.37 for each time point. The false significant number (FSN) ranged between 0.80 and 4.99, while the false discovery rate (FDR) ranged from 0.25 to 3.80. Of the 4708 *Xoo *strain MAI1 clones analysed, 710 genes were found to be differentially expressed with 407 up- and 303 down-regulated. The proportions of differentially expressed genes (up- or down-regulated) remained relatively constant over the first 3 days after inoculation but had changed markedly by day 6, with the proportions reversing (Table 1).

**Table 1:** Statistical summary of Significance Analyses of Microarrays (SAM)

Gene expression	Days after inoculation		
	1	3	6
Delta-delta Ct value	1.21	2.12	2.37
False significant number (FSN)	4.99	0.80	1.35
False discovery rate (FDR)	3.80	0.48	0.25
Up-regulated	58 (47%)	96 (40%)	253 (57%)
Down-regulated	66 (53%)	43 (60%)	194 (43%)

Total	124	239	447

The number of up- and down-regulated genes that are differentially expressed at different time points during infection by *Xanthomonas oryzae *pv. *oryzae*, African strain MAI1.

### Identification of differentially expressed genes

**Figure 2:**
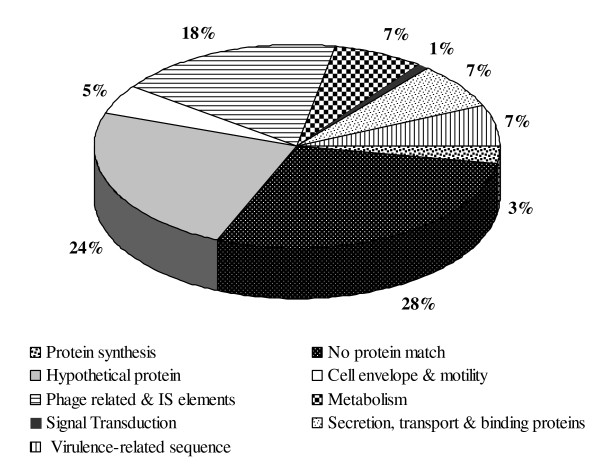
**Functional categorization of diferentially expressed genes**. Genes of *Xoo *strain MAI1 found as differentially expressed *in planta *were grouped into nine categories: biological process unknown; hypothetical protein; protein synthesis; cell envelope and motility; phage-related and IS elements; metabolism; signal transduction; secretion, transport, and binding proteins; and virulence-related sequence. The proportion of each category of the total number of genes is given as a percentage.

A total of 710 differentially expressed genes were one-end sequenced. After eliminating for low quality and vector contamination, 535 sequences were obtained. Insert size varied between 112 and 1902 bp, with an average of 660 bp. The initial data set of 535 good sequences was reduced to 147 unique consensus sequences, comprising 57 contigs and 90 singletons. To annotate the *Xoo *MAI1 non-redundant sequences, we used the Gene Ontology (GO) functional classification scheme [31]. Most functionally assigned non-redundant sequences (52%) fell into two classes: proteins with unknown function and biological process unknown (Figure 2). Mobile genetic elements, such as phage-related and IS elements, were well represented (18%). Secretion, transport, and binding proteins, together with virulence-related sequences, represented 14% of the differentially regulated genes (Figure 2).

### Thirty genes are specifically regulated

The set of 147 unique consensus sequences differentially expressed during infection, was searched against the genomes of all available sequenced strains of *X. oryzae *(*Xoo *strains KACC10331, MAFF311018, and PXO99^A^, and *Xoc *strain BLS256), and against the draft genome of the African *Xoo *strain BAI3. Results are summarized in the Additional file 1, Table S1. From these 147 genes, eight genes are present only in the African *Xoo *strains MAI1 and BAI3. Nine others are also only present in *Xoo *strains MAI1, BAI3, and PXO99^A^, and *Xoc *strain BLS256. Five are present only in *Xoo *strains MAI1 and BAI3, and *Xoc *strain BLS256 (Additional file 1, Table S1). Interestingly, a total of 30 *Xoo *MAI1 genes that were differentially expressed *in planta *are not present in the Asian *X. oryzae *genomes sequenced so far, indicating that these genes might be specific to the African *Xoo *strain MAI1. These MAI1-specific genes include 24 genes with no similarity to other proteins, two hypothetical proteins, one IS element, and three genes related to metabolism (Table 2).

**Table 2:** Differentially expressed genes that are specific to the African strain MAI1 of *Xanthomonas oryzae *pv. *oryzae *(*Xoo*)

GenBank accession	Library origin†	Seq. no. ‡	Putative function	Organism §	E-value	Size	**Time point**||			*Xanthomonas oryzae *genome¶				
							**1**	**3**	**6**	**MAFF 311018**	**KACC 10331**	**PXO 99A**	**BLS 256**	**BAI3**

**Biological Process Unknown**														
FI978294	1	1	No protein match (NPM)	-	-	1203	-			**-**	**-**	-	-	-
FI978293	1	1	NPM	-	-	974		+	+	**-**	**-**	-	-	-
FI978295	1	1	NPM	-	-	1233			+	**-**	**-**	-	-	-
FI978297	1	1	NPM	-	-	906			+	**-**	**-**	-	-	-
FI978298	1	1	NPM	-	-	975		+		**-**	**-**	-	-	-
FI978299	1	1	NPM	-	-	1499			+	**-**	**-**	-	-	-
FI978300	1	1	NPM	-	-	1122		-		**-**	**-**	-	-	-
FI978301	1	1	NPM	-	-	1659		+		**-**	**-**	-	-	-
FI978302	1	1	NPM	-	-	674	-		-	**-**	**-**	-	-	-
FI978303	1	1	NPM	-	-	1232			+	**-**	**-**	-	-	-
FI978101	1	1	NPM	-	-	409			+	-	-	-	-	-
FI978177	1	1	NPM	-	-	399			+	-	-	-	-	-
FI978197	1	1	NPM	-	-	248			-	-	-	-	-	-
FI978310	1	1	NPM	-	-	942			+	-	-	-	-	-
FI978308	1	1	NPM	-	-	931			+	-	-	-	-	-
FI978317	1	1	NPM	-	-	1175		+		-	-	**-**	**-**	**-**
FI978273	1	7	NPM	-	-	897			+	-	-	-	-	-
FI978320	1	1	NPM	-	-	1471			-	-	-	-	-	-
FI978321	1	1	NPM	-	-	1902			-	-	-	-	-	-
FI978086	1	1	NPM	-	-	544	-		-	-	-	-	-	-
FI978068	1	1	NPM	-	-	638	-	+	+	-	-	-	-	-
FI978327	2	1	NPM	-	-	876	-		-	-	-	-	-	-
FI978316	2	1	NPM	-	-	1157		+	+	-	-	-	-	-
FI978296	2	1	NPM	-	-	1529	+			-	-	-	-	-
FI978323	1	1	NPM	-	-	933			-	-	-	-	-	-
FI978322	2	1	NPM	-	-	861			+	-	-	-	-	-
**Hypothetical protein**														
FI978307	2	1	Hypothetical protein XCC2965	*Xcc *strain ATCC 33913	3.0E 12	835	-			-	-	-	-	-
FI978239	1 and 2	2	Hypothetical protein XCC2966	*Xcc *strain ATCC 33913	7.0E 11	244	+			-	-	-	-	-
**Phage-related and IS elements**														
FI978271	1	7	Gene transfer agent (GTA) like protein	*Parvibaculum lavamentivorans *strain DS 1	8.0E 50	788		+		-	-	-	-	-
**Metabolism**														
FI978324	1	1	Haemolysin III	*Xcc*	5.0E 17	853	-			-	-	-	-	-

†SSH library and/or libraries in which the clone was identified, where 1 corresponds to SSH library *Xoo *strain M1/PXO86, and 2 to SSH library *Xoo *strain M1/*Xoc *BLS256.

‡Number of sequences by contig, where 1 indicates singleton.

§*Xcc *is *Xanthomonas campestris *pv. *campestris; Xoo is Xanthomonas oryzae *pv. *oryzae*.

||Time point, in days after inoculation, where + indicates up-regulated, and - indicates down-regulated.

¶*Xanthomonas oryzae *genomes, where + indicates presence of gene homologues to Xoo MAI1 in the genome analysed, and - indicates absence.

We selected eight genes to validate their strain specificity, using Southern blot hybridization. These included two genes encoding for hypothetical proteins (FI978063 and FI978079), three genes encoding for proteins with unknown function (FI978168, FI978197 and FI978322), a probable secretion protein (FI978093) and two transposases (FI978069 and FI978109)(Additional file 1, Table S1). In all cases, DNA fragments were present in the *Xoo *MAI1 strain and absent from the corresponding DNA driver (*Xoo *PXO86 and/or *Xoc *BLS256) (data not shown). Additionally, we tested several other *X. oryzae *strains from different geographical origins, using FI978197 as probe, a fragment that is not shared between *Xoo *MAI1 and other *Xanthomonas *genomes (Table 2). Our findings showed that gene FI978197 was present only in *Xoo *strain MAI1 and absent in the other, both African and Asian, *Xoo *and *Xoc *strains (data not shown). Those genes corresponding to 'unknown function' may therefore represent interesting candidates for further functional analyses.

### Cluster analysis of microarray data

**Figure 3:**
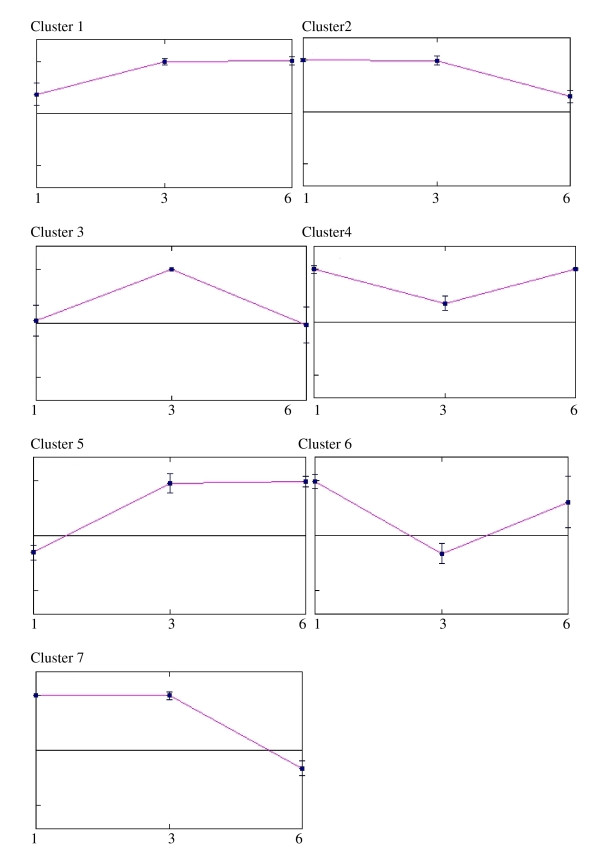
**Clusters of transcripts based on patterns of differential expression**. Differentially expressed transcripts were clustered, using the *k*-means method. The mean expression levels of genes in each cluster are shown as a centroid graph. Error bars represent standard deviations of expression within the cluster. Seven clusters were created, with clusters 1, 2, 3, and 4 comprising up-regulated genes and clusters 5, 6, and 7 comprising down-regulated genes at 1, 3, and 6 dai, respectively. The x axis represents time-points during infection (1, 3, and 6 dai) and the y axis the expression level.

A *k*-means clustering analysis was performed to obtain an overview of the performance of each differentially expressed gene, compared with the others during infection. Seven clusters were defined (Figure 3). Genes that were up-regulated were represented by clusters 1 (at 3 and 6 dai), 2 (1 and 3 dai), 3 (at 3 dai), and cluster 4 (at 1 and 6 dai). Down-regulated genes were represented by clusters 5, 6, and 7 at 1, 3, and 6 dai, respectively. Those differentially expressed genes in *Xoo *strain MAI1, which are discussed below as related to pathogenicity fell into these clusters.

### Activation of genes related to adhesion to plant system and plant cell-wall degradation during infection

*Xanthomonas oryzae *pv. *oryzae *is a vascular pathogen. A critical step in infection is adherence to the host's vascular surfaces [32]. Electron microscopy analysis during interaction between rice and *Xoo *showed bacterial cells within xylem vessels in both compatible and incompatible interactions after 1 dai [32]. Recently, the use of green fluorescent protein (GFP) technology showed that *Xoo *strain PXO99 _GFP _proliferated in susceptible rice lines but not in resistant lines at 12 dai [33].

Four genes fimbrial assembly protein (FI978267), pilin (FI978178), type IV pilin (FI978319), and the *pilY1 *gene (FI978318) that are associated with bacterial adhesion and biofilm formation were found as up-regulated in *Xoo *MAI1 *in planta *at 6 dai. These genes belong to cluster 1. Type IV pili are bacterial major virulence factors supporting adhesion, surface motility, and gene transfer [34-36]. The role of type IV pili genes in biofilm formation and virulence of phytopathogenic bacteria has been largely studied in the vascular pathogens *Ralstonia solanacearum *and *Xylella fastidiosa *[37-39] and, most recently, in *Xoo*, *Xac*, and *Xcc *[35,40,41]. Type IV in *Xoc *virulence increased with the presence of two *pilY1 *insertion mutants [42]. In *Xylella fastidiosa*, disruption of *pilY1 *reduced the number of type IV pili and the bacterium's capacity for twitching motility [43]. In *Xoo *and *Xoc*, grown on enriched medium, microarray analysis revealed the differential expression of several fimbrial assembly proteins [16]. Unlike the findings of previous studies which showed the presence of bacterial cells in xylem vessels after 12 hai [33], adherence-related genes were found to be induced later (cluster 1) in *Xoo *MAI1.

Biofilm formation and adherence capacities have been associated with virulence of pathogenic bacteria in *Xoo*, *X. axonopodis *pv. *citri *(*Xac*), *X. campestris *pv. *campestris *(*Xcc*), and others [35,36,40,44]. Inside plant tissues, biofilms are thought to contribute to virulence by blocking sap flow in the xylem vessels and promoting plant wilt [39]. The up-regulated genes involved in biofilm formation and pathogenicity were identified in *Xylella fastidiosa *through microarray analysis, which compared cells growing in a biofilm with planktonic cells [45]. In *Xoo *MAI1, we identified several of these genes as corresponding to type IV pili genes (e.g. FI978319) and the fimbrial assembly protein (e.g. FI978267) (Additional file 1, Table S1). Given that *Xoo*, like *Xylella fastidiosa*, is a restricted vascular pathogen, the induction of genes related to adhesion and motility suggests a role in biofilm formation and vascular colonization. The *Xoo *MAI1 strain regulates the expression of a group of genes for adherence and biofilm formation in the nutrient-limited environment of xylem in rice. This group's role in pathogenicity should be investigated.

Among the up-regulated genes in the *Xoo *MAI1 strain, we found one cellulase (FI978181) and one xylanase (FI978325) gene activated at 3 dai (cluster 1). Using an SSH approach, Qi et al. [46] identified the unique *Fibrobacter intestinalis *genes coding for plant cell-wall hydrolytic enzymes. More than 40 cellulases play a major role in *F. intestinalis *plant cell-wall degradation. An xylanase of *Xoo *was differentially expressed *in planta *[47]. Both enzymes (cellulase and xylanase) may play a similar role in *Xoo *MAI1 in degrading rice cell walls, thus facilitating pathogen multiplication.

### Major virulence genes are up-regulated *in planta*

Five classes of virulence genes were found regulated during infection. They corresponded to three genes related to the *avrBs3/pth *family (FI978282, M1P3I15, and AF275267), a leucin-rich protein (BAE68417), a virulence regulator (FI978260), and a *xopX *(ACD57163) and *hrpF *gene (FI978263). Most of these major virulence genes fell into cluster 1, corresponding to genes that are activated after 3 dai. *Xoo *pathogenicity is highly dependent on the type III secretion system (TTSS) injecting effector proteins into the eukaryotic host cell [48]. Most knowledge on TTSS in *Xoo *is based on studies of the large AvrBs3/pthA family of *Xanthomonas *effector proteins [49]. This family includes proteins with avirulence activities, virulence functions, or both [48]. It includes the well-characterized AvrXa7 protein, which plays a role in bacterial growth and lesion development in rice [50,51]. Genes *avrXa7 *(AF275267) and *xopX *(ACD57163) are up-regulated at both 3 and 6 dai. The *xopX *gene encodes a TTSS effector protein and contributes to the virulence of *X. campestris *pv. *vesicatoria *on hosts pepper and tomato [52]. XopX targets the innate immune response, resulting in enhanced plant disease susceptibility [52]. The XopX protein from *Xcc *is required for full virulence, as shown by the *XccN *mutant that produced weaker disease symptoms than the wild-type strain [53].

The HrpF protein is probably inserted into the plant-cell membrane and may be required for the bacterium's type III effector proteins to enter host cells [54]. As a bacterial translocon, HrpF would therefore be in direct contact with the plant-cell membrane and even possibly subjected to the plant's surveillance mechanisms while it mediates effector protein delivery across the host-cell membrane. To demonstrate that HrpF is required for pathogenicity, Sugio et al. [55] used *Xoo hrpF *mutants, which had a reduced ability to either grow within rice plants or cause lesions. For the *Xoo *MAI1 strain, we found a *hrpF *gene that was differentially expressed at 3 dai during infection. The activation of different genes encoding proteins secreted by TTSS (*hrpF*, *avrXa7*, and *xopX *genes) during *Xoo *MAI1-rice interaction was consistent with TTSS being essential for *Xoo *pathogenicity.

### Expression of IS elements in *Xoo *MAI1 during infection

Insertion sequence (IS) elements have recently been shown to play a role in plant pathogenicity [56-59]. These elements may inactivate genes on insertion or activate and/or enhance the expression of nearby genes [57,60,61]. One characteristic of the *Xoo *genomes sequenced to date is the accumulation of many IS elements, representing as much as 10% of the *Xoo *genome size [23]. In *Xanthomonas *spp., virulence and pathogenicity islands are commonly associated with mobile genetic elements such as phages and transposons [56,58]. By comparing gene expression of both *Xoo *and *Xoc *grown in enriched versus minimal medium, Seo et al. [16] determined that IS elements are differentially expressed in minimal medium.

In our study, we identified 27 IS elements in *Xoo *MAI1 that are up- or down-regulated *in planta*. Most of these IS elements belong to cluster 1, corresponding to genes that are activated after 3 dai. Twelve elements were classified into the following IS families: IS*30 *(4 elements), IS*5 *(7), and IS*3 *(1), with 15 IS elements unclassified. Members of the IS*5 *family have been reported previously in bacterial pathogens and it has been speculated that expression of some pathogenicity genes might be controlled by the expression/insertion of IS5 family elements [58,62,63]. Expression of IS5 members in the neighboring region of their *hrp *gene cluster was observed in *Pseudomonas syringae *[64] It has been also demonstrated that IS elements (among them some IS5 family members) can act as a mobile switch for the downstream genes, creating new transcriptional promoters and increasing the expression levels of downstream genes [65]. Members of the IS*3 *and IS*30 *families have also been reported in bacterial pathogens, some of them controlling the expression of other genetic elements [60,66]. The expression of IS elements in *Xoo *MAI1 *in planta *suggests that these elements may play a significant role in bacterial pathogenicity or may be associated with genes related to pathogenicity.

To establish a correlation between the presence of IS elements and adjacent genes differentially expressed in MAI1, we used the draft genome of *Xoo *African strain BAI3 (Genoscope project 154/AP 2006-2007 and our laboratory, 2009, unpublished data) and the published genome of *Xoo *strain MAFF311018 [22]. We compared the location of the 147 *Xoo *MAI1 differentially expressed genes with the presence of adjacent IS elements in the *Xoo *BAI3 and MAFF311018 genomes. For this, homologous sequences of IS elements, found as differentially expressed in the *Xoo *strain MAI1, were first identified in the BAI3 draft genome. We then extracted 10 kb from each of up- and downstream flanking regions of IS elements. BLAST searches were performed against these flanking regions, using the *Xoo *MAI1 non-redundant set of sequences. For the sequences located within 20 kb of sequences flanking the IS elements, we compared the relative distance of each sequence to the IS element in BAI3 with the relative distance of their respective homologues in the *Xoo *MAFF311018 genome (Table 3).

**Table 3:** Homologues of genes in strain MAI1 found near IS elements in the BAI3 genome

			Relative distance (kb) between differentially expressed genes and IS elements in genome:	
Flanking sequence of IS element	Genes in vicinity	Putative function	BAI3	MAFF311018
**FI978233**			10001..10132	920135..920004
ISXo8 transposase (IS5 family)	FI978262	ISXo8 transposase (IS5 family)	- 2.0	- 3723
	FI978083	Putative transposase	+ 8.2	+ 621
	M1P4B2	No protein match	+ 1.2	- 3796
	FI978246	Transposase	+ 6.3	- 1089
	FI978279	ribonucleoside-diphosphate reductase, beta subunit	- 0.9	+ 153
	FI978268	No protein match	+ 7.8	+ 761
	FI978290	dTDP-glucose 4,6-dehydratase	- 10.1	+ 144
	FI978285	hypothetical protein XOO1934	- 1.2	+ 150
	FI978270	Putative transposase	- 3.8	+ 757
**FI978246**			10001..10299	2009657..2009789
transposase	FI978181	Cellulase	- 5.5	+ 1728
**FI978274**			13384..14161	14199973..1420912
ISXoo15 transposase (IS30 family)	FI978084	Putative transposase	+ 7.8	+13.8

Homologues of IS elements, found as differentially expressed in the African strain MAI1 of *Xanthomonas oryzae *pv. *oryzae *(*Xoo*) were identified in the *Xoo *BAI3 draft genome. Extractions (10 kb) were made from up- and downstream flanking regions of IS elements. BLAST searches were performed locally, using the MAI1 differentially expressed genes. For the sequences located within the 20-kb sequence flanking the IS elements, the relative distance of each sequence to the IS element in BAI3 was compared with the relative distance of their respective homologues in the *Xoo *MAFF311018 genome. The designation + indicates upstream location of the sequence relative to the IS element, and the designation - indicates downstream location. For IS elements, gene locations within the 20-kb sequence flanking the IS element in BAI3 and within the genome of *Xoo *MAFF311018 are presented.

Results showed that homologues of the 11 selected *Xoo *MAI1 differentially expressed genes are located in the vicinity of IS elements in BAI3 genome, within the same 20-kb region (Table 3). In the *Xoo *MAFF311018 genome, *Xoo *MAI1 differentially expressed genes are not located in a vicinity of 20 kb of the IS elements. Given that the African *Xoo *strain BAI3 is more closely related to *Xoo *MAI1 than *Xoo *MAFF311018, a similar organization of IS elements and presence of neighbour genes are expected for MAI1. Correlation between differential expression of IS elements, genome location, and role played in the control of expression of nearby genes in African *Xoo *strains need further study.

### Validation of differentially expressed genes, using QRT-PCR

**Figure 4:**
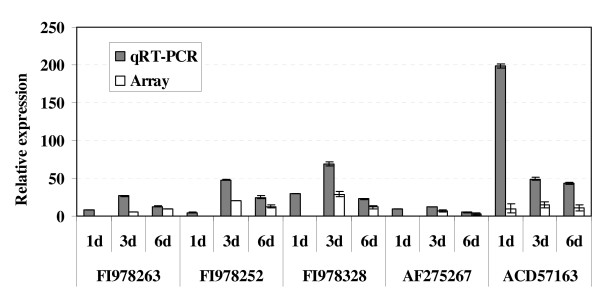
**Comparing expression of genes through microarray and QRT-PCR assays**. We used real-time PCR analysis to confirm the differential expression of 14 genes of the African strain MAI1 of *Xanthomonas oryzae *pv. *oryzae*. The genes represented various biological functional classes of interest. Although fold change in gene expression was generally higher for QRT-PCR than for the microarray, good correlation existed between the two data sets. A subset of 5 genes corresponding to *xopX *(ACD57163), HrpF protein (FI978263), hypothetical proteins (FI978252 and FI978328), and the *avrXa7 *gene (AF275267) showing a fold-change higher than 10 in QRT-PCR is illustrated in the figure. Three replicates were analysed in both microarray and QRT-PCR experiments. Vertical bars represent standard deviations.

To validate the *Xoo *MAI1 microarray results, QRT-PCR was performed on a set of 14 genes of different functions and which were up- or down-regulated during infection. Table 4 lists the primers, putative function, and average fold-change expression of genes used for QRT-PCR validation. The genes selected for QRT-PCR correspond to four hypothetical proteins (FI978067, FI978252, FI978305, and FI978328), one gene showing no similarity to known proteins (FI978310), two putative transposases (FI978288 and FI978099), two genes related to transport and motility (FI978259 and FI978319), one *hrpF *gene (FI978263), and one avirulence protein from the AvrBs3/pthA family (F1978282), the *avr/pth14 *gene (M1P3I15), the *xopX *gene (ACD57163), and the *avrXa7 *gene (AF275267). Figure 4 shows five genes out of the 14 tested that were up-regulated by QRT-PCR and having a larger than 4-fold change. Of the 14 genes selected according to the microarray data (Table 4), 13 were up-regulated and 1 (F1978067) was down-regulated. The QRT-PCR results supported these data, and also showed that the gene expression pattern was identical for all genes tested, except two (FI978259 and FI978319). Gene expression values, however, differed between microarrays and QRT-PCR. As shown in Figure 4, the expression values for the five genes FI978252, FI978263, FI978328, AF275267, and ACD57163 were higher in QRT-PCR than for microarray, indicating that QRT-PCR may be more sensitive than microarray analysis. The *xopX *gene (ACD57163) was highly up-regulated in *Xoo *strain MAI1 *in planta*, indicating that induction of this gene is important during interaction between *Xoo *strain MAI1 and rice. These five genes belonged to cluster 1.

**Table 4:** Validation by QRT-PCR of differentially expressed genes

								Fold changes in gene expression		
								
					Array			QRT-PCR		
					
Gene	Putative function	Primer sequence	Size, bp	AT, °C	1 dpi	3 dpi	6 dpi	1 dpi	3 dpi	6 dpi
**FI978319**	Type IV pilin	5' CTAACCGGCTGAGCTATTCG	166	60	0,0	0,0	1,3	2,7	2,9	2,0
		3' CAGCCAAGCCAAAGACAAGT								
**FI978328**	probable TonB-dependent receptor	5' CGCACTAATCGCATTCTCAA	167	60	0,0	29,0	11,7	29,9	69,0	22,5
		3' AAACGGCGGATGTAGAACAG								
**FI978288**	putative transposase	5' GCAGAACGTTGGGAACACTT	156	60	0,0	1,7	0,8	0,5	0,4	1,0
		3' CAGATTCGACAGCGCAAATA								
**FI978282**	avirulence protein AvrBs3/pth family	5' AAGAGGAACTCGCATGGTTG	167	60	0,0	0,6	1,3	0,7	0,6	1,6
		3' TTGAACGCATCTGTCTACCG								
**FI978099**	putative transposase	5' TCGTTTTGTTAGCCGCTCTT	188	60	0,0	0,9	1,4	0,0	0,8	1,6
		3' GACGCACATTGCACTTTGAT								
**M1P3I15**	Avr/Pth14 (avr/pth14) gene	5' AGGTACGAGGCCTCACTGAA	140	60	0,0	1,4	1,9	3,2	3,4	8,1
		3' CAATTCCCTATCCCGAGGAG								
**FI978263**	HrpF protein	3' GGGCTAACAATCACCAGAGC	157	60	0,0	5,0	9,8	8,3	26,7	12,5
		5' CACGTTTTCGGGATTCAAGT								
**FI978252**	hypothetical protein XOO0776	5' AGAAGTTGCAGGCCAAAGAA	150	60	0,0	20,0	12,3	4,3	47,5	24,9
		3' CGCAGGTGACAAACAAAAGA								
**FI978310**	-	5' AATGGATCAGTTGGGTTGGA	224	60	0,0	0,0	1,5	0,0	1,2	1,1
		3' GAGGTACGCTtcgaCGTTTC								
**FI978259**	ATP-binding protein of ABC transporter	5' TCAGCTCATTTCACGTCAGG	215	60	0,0	0,0	1,6	2,5	1,7	1,6
		3' CAGAGCAGGGTGTGTAAGCA								
**FI978067**	conserved hypothetical protein	5' GCATATAGCTCCGAGGCAAC	160	60	0,0	-2,2	0,0	-0,8	-2,8	-0,2
		3' GGTTTCCCCATTCGGATATT								
**FI978305**	hypothetical protein xccb100_3708	5' AGGAGCCAAGGCAATTAACA	170	60	0,0	0,0	0,5	0,5	0,6	1,2
		3' TGAGGAGTCTGGGAAGTTGG								
**ACD57163**	XopX effector protein	5' TTGTTCCTGCCATTGGAAAT	150	60	10,0	14,7	11,0	198,5	49,0	43,3
		3' GATGCTGCTCCAGAGAAAGG								
**AF275267**	avirulence protein gene (avrXa7)	5' GCACAGCAATCTTTCGAGGT	172	60	0,0	7,2	3,0	9,8	12,3	4,8
		3' CATCTTGTTCCCACATCACG								

List of DNA fragments used to validate the *Xanthomonas oryzae *pv. *oryzae *(*Xoo*) MAI1 strain expression changes as determined by microarray analysis. Sequences of forward and reverse primers, putative function; average of fold-change expression, gene product sizes, and annealing temperatures (AT) are indicated.

## Conclusions

Sustainable control measures for bacterial blight in Africa will depend on understanding and characterizing those of the microbe's genes involved in the rice-*Xoo *interaction. We therefore focused our study on analysing and characterizing *Xoo *MAI1 at the transcriptional level. For this we constructed a *Xoo *MAI1 SSH array, performed *in planta *gene expression analysis and selected and validated by QRT-PCR various gene expressions to generate robust and reliable data. Although the SSH microarray may not be as sensitive as QRT-PCR for some genes, results included several candidate genes whose regulation and function will need to be elucidated to better understand the *Xoo*-rice interactions.

Our study shows that the regulation of gene expression in the *Xoo *strain MAI1 is controlled at different time points during pathogen infection. We identified conserved mechanisms for which some were reported in other *Xoo*-plant interactions but not yet described for African strains. We also identified differentially regulated genes specific to the *Xoo *strain MAI1. Several homologues of *Xoo *MAI1 differentially expressed genes were located in the vicinity of IS elements in the *Xoo *BAI3 genome. The role played by these IS elements in controlling neighbouring-gene expression needs to be elucidated. More data on African *Xoo *strains also need to be generated. Recently, the sequencing of various African *Xoo *and *Xoc *strains has been initiated at our laboratory and others. With this information, the full-length cDNA of desired genes can be easily obtained and their specific functions in pathogenicity studied, using available gene knockout technology. Functional characterization of the proteins/genes related to virulence will be of particular importance in understanding the complex interaction between *Xoo *MAI1 and rice. Our work constitutes a significant contribution towards the biology of an emerging and devastating pathogen under a specific, but insufficiently studied, environment in West Africa.

## Methods

### DNA microarray construction

Two subtracted DNA libraries (SSH) were previously constructed in our laboratory and partially characterized [28]. For the first library (MAI1-PXO86), the tester was the African *Xoo *MAI1 (race A3) and the driver the Philippine *Xoo *PXO86 (Phil race 2). For the second library (MAI1-BLS256), the same tester was used, with *Xoc *BLS256 being the driver. We randomly selected 2112 clones from MAI1-PXO86 library and 2304 from MAI1-BLS256. From the MAI1-PXO86 SSH library, we selected another 88 clones that represented a non-redundant set of sequences selected from a previous analysis of 265 sequences from that library [28]. Several controls (clones from genes of other bacteria, fungi, rice, and humans) were also included. Inserts from each DNA clone were PCR-amplified directly from bacteria. Amplification reactions were performed in 96-well plates, with each well carrying a 50-μl volume containing 0.2 μM of each primer (T7 and SP6), 200 μM of each dNTP, 1× PCR buffer, and 1.25 units of *Taq *polymerase (AmpliTaq^® ^DNA polymerase, Promega Corporation). An MJ Research thermal cycler was used for 35 PCR cycles, as follows: 95°C for 45 s, 56°C for 45 s, and 72°C for 1 min. We also amplified a selected set of conserved effector and *hrp *genes (e.g. *XopX*, *avrXa7*, *XopD*, *avrRxv*, *avrXv3*, *hpaF*, and *hrpx*), housekeeping genes, and other conserved bacterial genes from genomic DNA of *Xoo *MAI1. Random PCR samples were visualized on agarose gels. All PCR products were transferred to a 384-well plate and a volume of 2× betaine solution was added. The PCR products were arrayed once on poly-L-lysine slides (TeleChem International, Inc., Sunnyvale, CA, USA), using an SPBIO™ Microarray Spotting Station (MiraiBio, Inc., Alameda, CA, USA). The microarray contained 4708 elements.

### Bacterial inoculation and quantification

The *Xoo *strain MAI1 was grown on PSA medium (10 g l^-1 ^peptone, 10 g l^-1 ^sucrose, 1 g l^-1 ^glutamic acid, 16 g l^-1 ^agar, and pH 7.0) for 2 days at 30°C. The bacterial cells were re-suspended in sterilized water at an optical density of 600 nm (OD _600_) (about 10^-9 ^cfu ml^-1^). Bacterial blight inoculation was carried out on the two youngest, fully expanded leaves on each tiller of 6-week-old rice plants (var. Nipponbare), using the leaf-clipping method [67]. Experiments were conducted under greenhouse conditions at 26°C and 80% relative humidity.

We determined *Xoo *MAI1 multiplication *in planta *at seven time points after infection by leaf clipping (0 and 12 h, and 1, 3, 6, 10, and 15 days after inoculation) in 8-week-old plants of the susceptible rice cultivar Nipponbare. The number of cells in the leaves was determined at the top 10 cm of each leaf which was cut into five 2-cm sections, and labelled A, B, C, D, and E, with A being the inoculation point. The leaf pieces were then ground in 1 ml of sterilized water. Serial dilutions were made and spread onto PSA agar plates. The plates were incubated at 28°C until single colonies could be counted. The number of colony-forming units (cfu) per leaf (equivalent to about 2 cm^2^) was counted and standard deviations calculated. The experiment was repeated independently three times.

### RNA extraction

To obtain RNA from cells growing *in planta*, 30 rice leaves were inoculated by the leaf-clipping method. At each time point, leaves extending 2 cm from the tip were collected and, to facilitate exudation of bacterial cells, vortexed for 30 s with RNAprotect Bacteria Reagent (QIAGEN, Inc., Courtaboeuf, France). The leaves were removed and bacterial cells were collected in a 15-ml tube by centrifuging at 4000 rpm for 30 min at 4°C. Total RNA extraction and DNase treatment were conducted, using the RNeasy Mini Kit according to the manufacturer's recommendations (QIAGEN, Inc.). To obtain RNA from bacterial cells, bacterial cultures were grown on PSA medium at 28°C until the early stationary phase. They were then re-suspended in 15 ml sterilized Milli-Q water, adjusted to OD _600 _of 0.2 (about 10^-8 ^cfu ml^-1^), pelleted by centrifuging, and transferred to 1.5-ml tubes. Total RNA and DNase I treatments were performed as described above. The RNA quality was verified both by agarose-gel electrophoresis and by PCR (for presence of genomic DNA), using the genomic region flanking the *hrpX *gene as control and purified RNA as the PCR template. About 1 μg of *Xoo *MAI1 total RNA, obtained from cells grown in culture medium or *in planta *and treated with DNase I, were used individually to synthesize single-stranded cDNA. The SMART™ PCR cDNA Synthesis Kit (BD Biosciences Clontech) was used, following the manufacturer's instructions. The cDNA was then quantified, using the PicoGreen^® ^reagent (Invitrogen, Ltd., Paisley, UK), an ultra-sensitive, fluorescent, and nucleic dye.

### DNA microarray hybridization

Fluorescent-labelled probes were prepared, following the Klenow labelling method (indirect labelling). Briefly, 500 ng of cDNA were labelled, using 1 μl of either Cy3- or Cy5-dUTP (Amersham Pharmacia Biotech, Little Chalfont, UK), 10 U Exonuclease-Free Klenow (USB Corporation, Cleveland, OH, USA), and 3 μg random primers (Invitrogen Life Technologies, Carlsbad, CA, USA), and incubated 2 h at 37°C. Unincorporated nucleotides were removed, using a QIAquick PCR Purification Kit (QIAGEN, Inc.). Cleaned probes were concentrated in a speedvac (Eppendorf^® ^Vacufuge Concentrator 5301, Hamburg, Germany).

Before hybridization, glass slides were snap-dried on a 95°C heating block for 10 s. DNA was crosslinked to the slides, using 65 mJ of 254-nm UV-C radiation from a Stratalinker^® ^UV Crosslinker (model 2400, Stratagene, La Jolla, CA, USA). Slides were incubated 2 h at 70°C and pre-hybridized with 1% BSA, 5× SSC buffer, and 0.1% (w/v) SDS for 45 min at 54°C. The hybridization mixture consisted of 500 ng labelled cDNA and 4.5 μg μl^-1 ^of salmon sperm DNA (Invitrogen Life Technologies) in a final volume of 35 μl. This volume was mixed with 35 μl of 2× hybridization buffer (1× formamide, 1× SSC, and 0.04× SDS). The mixture was denatured at 95°C for 2 min and transferred to ice. The hybridization mixture was applied to a microarray slide, transferred immediately to a hybridization chamber (Corning, Inc., Lowell, MA, USA), and incubated overnight (15-17 h) at 42°C. The slide was then washed for 5 min successively in each of 2× SSC, 0.1% (w/v) SDS at 54°C, 1× SSC, and 0.1× SSC at room temperature. Slides were immediately dried by centrifuging at 1000 rpm for 4 min.

At each time point, cDNA, obtained from bacteria used as inoculum and re-suspended in water (time 0), was compared with bacteria recovered from inoculated plants at 1, 3, and 6 dai. The comparisons made were 0 - 1, 0 - 3, and 0 - 6 dai. To maximize the statistical reliability of the data, three biological replicates were carried out.

In addition, for each time point comparison and each biological replicate, three technical replicates (cDNA obtained from the same mRNA extraction) were used for hybridization. For one of the three technical replicates, the labelling of the two cDNA samples with either Cy5 or Cy3 fluorescent dye was reversed to prevent potential dye-related differences in labelling efficiency. Overall, 27 images were analysed, 9 for each time point during *Xoo *infection. The nine data points obtained for each gene were used in the analyses.

### Microarray data analysis

The slides were scanned, using a chip reader/scanner (Virtek Vision International, Inc., Waterloo, ON, Canada). The signal was initially normalized during image scanning to adjust the average ratio between the two channels, using control spots. Spot intensities from scanned slides were quantified, using the Array-Pro 4.0 software (Media Cybernetics, Inc., Silver Spring, MD, USA). With this program, local corner background correction was carried out. Array-Pro 4.0 output data files (in Excel) were used to perform the lowest intensity normalization, standard deviation regularization, low intensity filtering, and dye-swap analysis, using the MIDAS computer program [68]. Normalization between different slides was carried out by centring [69]. MIDAS [68] was also used for replicate analysis and dye-swap filtering.

Bootstrap analyses with SAM enabled us to identify the differentially expressed genes, using a cut-off of two and adjusting the delta-delta Ct value, FDR, and FSN to minimize the number of false positives genes [70]. We conducted *k*-means clustering analysis to group the cDNA clones according to the similarity of their expression patterns, using MeV software available from TIGR and the default options [68].

### Sequence data analysis

The 710 genes identified as differentially expressed were one-end sequenced. Sequence data were processed, using a PerlScript pipeline, to remove vector and low-quality sequences and to assemble sequences into a non-redundant set of sequences [71]. The *Xoo *MAI1 non-redundant set of sequences was deposited at GenBank's GSS Database http://www.ncbi.nlm.nih.gov/dbGSS/[72], under accession numbers FI978231-FI978329.

Processed sequences were initially searched against the NCBI database with BLASTN and TBLASTX http://blast.ncbi.nlm.nih.gov/Blast.cgi[73], setting BLAST parameters to search against the complete non-redundant database and the genomes of *Xoo *strains KACC10331, MAFF311018, and PXO99^A^, and *Xoc *strain BLS256. A BLAST search was also performed with the partial genome of the African *Xoo *strain BAI3, which is currently being sequenced (Genoscope project 154/AP 2006-2007 and our laboratory, 2009, unpublished data). Results of these comparisons are summarized in the Additional file 1, Table S1. The *Xoo *MAI1 non-redundant set of sequences was grouped into functional categories, using Gene Ontology (GO), a functional classification scheme http://www.geneontology.org[74].

To establish if differentially expressed genes are located in the vicinity of the IS elements in the genomes of *Xoo *African strain BAI3 and *Xoo *Asian strain MAFF311018, we selected a region of 20 kb that flanked the IS elements in both the MAI1 and BAI3 genomes. BLAST searches were performed against these flanking sequences, using the *Xoo *MAI1 non-redundant set of sequences. For the sequences located within the 20-kb sequence flanking the IS elements, the relative distance of each sequence to the IS element was calculated and compared between the two genomes.

### Southern blot analysis of differentially expressed genes

Southern blot analysis was used to confirm that the DNA fragments derived from individual clones were present in the initial tester (*Xoo *MAI1 strain) and absent in the driver DNA (*Xoo *PXO86 or *Xoc *BLS256 strain). Eight genes (FI978063, FI978069, FI978079, FI978093, FI978109, FI978168, FI978197 and FI978322) were selected according to sequence similarities and library origin. Additionally, the gene FI978197 was selected to screen genomic DNA from different *Xoo *Asian strains (HN35, PXO339, PXO341, and PXO86), *Xoo *African strains (MAI1, BAI3, NAI8, and BAI4), *Xoc *African strains (MAI11 and MAI3), and the *Xoc *Asian strain BLS256 (Figure 2).

Briefly, for each strain, 5 μg of genomic DNA was digested with 10 units of *Rsa*I and run on 0.8% agarose gels. The DNA was transferred to Hybond-N^+ ^nylon membranes (Amersham Pharmacia Biotech) by capillary transfer. The insert DNA was amplified by PCR, using the nested primers provided with the PCR-Select™ Bacterial Genome Subtraction Kit (Clontech Laboratories, Inc.). The amplified DNA fragment was gel purified, using the QIAquick Gel Extraction Kit (QIAGEN, Inc.), as recommended by the manufacturer. The DNA fragments were labelled with [α^32^P] dCTP by random priming (MegaPrime labelling kit, Amersham Biosciences). Conditions of hybridization and washes were done at 65°C. Filters were washed with three solutions: the first of 2× SSC and 0.1% SDS for 20 min, followed by two washings with 1× SSC and 0.1% SDS for 10 min each, and a final wash with 0.1× SSC and 0.1% SDS for 20 min. Blots were exposed on a PhosphorImager (model Storm 860, Amersham Pharmacia Biotech Inc.-Molecular Dynamics Division, Piscataway, NJ, USA).

### Validation by quantitative QRT-PCR

We selected 14 genes that had been differentially expressed at various time points during infection by *Xoo *MAI1 for confirmation by QRT-PCR. The primers for quantitative detection were designed, using the Beacon Designer™ software (PREMIER Biosoft International, Palo Alto, CA, USA) (Table 4). All experiments were performed in triplicate. PCR mixtures were prepared, using FullVelocity^® ^SYBR^® ^Green QPCR Master Mix (Stratagene). PCR was performed on an Mx3005P thermal cycler (Stratagene), with the following cycling program: 95°C for 5 min, followed by 40 cycles of 10 s at 95°C and 30 s at 60°C.

Analysis was performed, using the delta-delta Ct method. The gene expression levels obtained by QRT-PCR were normalized, using the 16S ribosomal gene, which showed similar expression levels at different time points after infection. The gene expression level was compared between microarray and QRT-PCR. Similarly, the microarray ratio for each gene analysed was normalised against the microarray ratio obtained for 16S ribosomal gene. This allowed direct comparison between the 16S ribosomal gene-normalized QRT-PCR ratio and the 16S ribosomal gene microarray ratio for each transcript investigated.

## Authors' contributions

MS JT and VV designed the research project. MS DB and CG constructed the SSH, prepared samples for microarray studies and performed the microarray experiments. MS and DB analyzed microarray data. MS and RG carried out sequence analysis, MS and BS designed QRT-PCR experiments. MS and VV drafted the manuscript. All authors read and approved the final manuscript.

## Supplementary Material

Additional file 1 ***Xoo *strain MAI1 genes identified as differentially expressed *in planta *by microarray analysis**. The non-redundant set of sequences, composed of 147 *Xoo *strain MAI1 genes differentially expressed during infection, was searched against the genomes of all available sequenced strains of *X. oryzae *(*Xoo *strains KACC10331, MAFF311018, and PXO99^A^, and *Xoc *strain BLS256), and against the draft genome of the African *Xoo *strain BAI3. Changes in gene expression across different time points during infection are also presented. Click here for file
