# Supplementary material for: In planta gene expression analysis of Xanthomonas oryzae pathovar oryzae, African strain MAI1
Source: BMC Microbiol. 2010 Jun 11;10:170. doi: 10.1186/1471-2180-10-170 (PMC2893596; doi:10.1186/1471-2180-10-170)
Supplement: Additional file 1 — Xoo strain MAI1 genes identified as differentially expressed in planta by microarray analysis. The non-redundant set of sequences, composed of 147 Xoo strain MAI1 genes differentially expressed during infection, was searched against the genomes of all available sequenced strains of X. oryzae (Xoo strains KACC10331, MAFF311018, and PXO99A, and Xoc strain BLS256), and against the draft genome of the African Xoo strain BAI3. Changes in gene expression across different time points during infection are also presented. [file 1471-2180-10-170-S1.DOC]

**Table S1.** *Xoo* strain MAI1 genes identified as differentially expressed *in planta* by microarray analysis.

| **GenBank_Accn** | **Library origin**† | **N. seq‡** | **Putative function** | **Organism**†† | **e-value** | **Size** | **Timepoints**∥ | | | ***Xanthomonas oryzae* genome**¶ | | | | |
| --- | --- | --- | --- | --- | --- | --- | --- | --- | --- | --- | --- | --- | --- | --- |
|  |  |  |  |  |  |  | **1d** | **3d** | **6d** | **MAFF 311018** | **KACC10331** | **PXO99A** | **BLS256** | **BAI3** |
| **Protein synthesis** |  |  |  |  |  |  |  |  |  |  |  |  |  |  |
| FI978291 | 1 | 1 | translation elongation factor Tu | *Xoo* PXO99A | 7.0E-16 | 836 |  |  | + | + | + | + | + | + |
| FI978292 | 1 | 1 | 30S ribosomal protein S8 | *Xcc* str. ATCC 33913 | 5.0E-41 | 821 |  | - | + | + | + | + | + | + |
| FI978280 | 2 | 12 | rRNA-23S ribosomal RNA | *Xoo* PXO99A | 0 | 557 | - |  |  | + | + | + | + | + |
| FI978284 | 1 and 2 | 22 | rRNA-23S ribosomal RNA | *Xoo* KACC10331 | 0 | 535 | + |  |  | + | + | + | + | + |
| **Biological process unknown** |  |  |  |  |  |  |  |  |  |  |  |  |  |  |
| FI978294 | 1 | 1 | No protein match (NPM) | - | - | 1203 | - |  |  | **-** | **-** | - | - | - |
| FI978293 | 1 | 1 | NPM | - | - | 974 |  | + | + | **-** | **-** | - | - | - |
| FI978295 | 1 | 1 | NPM | - | - | 1233 |  |  | + | **-** | **-** | - | - | - |
| FI978297 | 1 | 1 | NPM | - | - | 906 |  |  | + | **-** | **-** | - | - | - |
| FI978298 | 1 | 1 | NPM | - | - | 975 |  | + |  | **-** | **-** | - | - | - |
| FI978299 | 1 | 1 | NPM | - | - | 1499 |  |  | + | **-** | **-** | - | - | - |
| FI978300 | 1 | 1 | NPM | - | - | 1122 |  | - |  | **-** | **-** | - | - | - |
| FI978301 | 1 | 1 | NPM | - | - | 1659 |  | + |  | **-** | **-** | - | - | - |
| FI978302 | 1 | 1 | NPM | - | - | 674 | - |  | - | **-** | **-** | - | - | - |
| FI978303 | 1 | 1 | NPM | - | - | 1232 |  |  | + | **-** | **-** | - | - | - |
| FI978236 | 1 | 1 | NPM | - | - | 252 |  |  |  | **-** | **-** | + | + | + |
| FI978234 | 1 | 1 | NPM | - | - | 850 |  |  |  | - | - | - | + | + |
| FI978244 | 1 | 2 | NPM | - | - | 849 |  |  | - | - | - | - | + | + |
| FI978268 | 1 | 6 | NPM | - | - | 266 |  |  | - | + | + | + | + | + |
| FI978101 | 1 | 1 | NPM | - | - | 409 |  |  | + | - | - | - | - | - |
| FI978177 | 1 | 1 | NPM | - | - | 399 |  |  | + | - | - | - | - | - |
| FI978197 | 1 | 1 | NPM | - | - | 248 |  |  | - | - | - | - | - | - |
| FI978168 | 1 | 1 | NPM | - | - | 128 |  |  | - | - | - | + | - | - |
| FI978092 | 1 | 1 | NPM | - | - | 619 |  |  | - | - | - | - | - | + |
| M1PG05BGS2 | 1 | 1 | NPM | - | - | 85 |  | + | + | + | + | + | + | + |
| FI978077 | 1 | 1 | NPM | - | - | 135 | - | + | + | - | - | + | + | + |
| FI978163 | 1 | 1 | NPM | - | - | 145 |  | + |  | - | - | + | - | + |
| M1P4B2 | 1 | 1 | NPM | - | - | 112 |  | + |  | - | - | + | - | + |
| FI978179 | 1 | 1 | NPM | - | - | 369 | - |  |  | + | + | + | + | + |
| FI978172 | 1 | 1 | NPM | - | - | 203 | - |  |  | + | + | + | + | + |
| FI978158 | 1 | 1 | NPM | - | - | 345 | - |  |  | + | + | + | + | + |
| FI978311 | 1 | 1 | NPM | - | - | 903 |  |  | + | - | - | - | - | - |
| FI978281 | 1 | 12 | NPM | - | - | 464 |  |  | - | - | - | + | + | - |
| FI978314 | 1 | 1 | NPM | - | - | 720 |  |  | + | - | - | + | + | - |
| **FI978310** | 1 | 1 | NPM | - | - | 942 |  |  | + | - | - | - | - | - |
| FI978308 | 1 | 1 | NPM | - | - | 931 |  |  | + | - | - | - | - | - |
| FI978317 | 1 | 1 | NPM | - | - | 1175 |  | + |  | - | - | **-** | **-** | **-** |
| FI978273 | 1 | 7 | NPM | - | - | 897 |  |  | + | - | - | - | - | - |
| FI978320 | 1 | 1 | NPM | - | - | 1471 |  |  | - | - | - | - | - | - |
| FI978321 | 1 | 1 | NPM | - | - | 1902 |  |  | - | - | - | - | - | - |
| FI978086 | 1 | 1 | NPM | - | - | 544 | - |  | - | - | - | - | - | - |
| FI978070 | 1 | 1 | NPM | - | - | 478 |  |  | - | - | - | - | - | + |
| FI978068 | 1 | 1 | NPM | - | - | 638 | - | + | + | - | - | - | - | - |
| FI978327 | 2 | 1 | NPM | - | - | 876 | - |  | - | - | - | - | - | - |
| FI978316 | 2 | 1 | NPM | - | - | 1157 |  | + | + | - | - | - | - | - |
| FI978296 | 2 | 2 | NPM | - | - | 1529 | + |  |  | - | - | - | - | - |
| FI978323 | 1 | 1 | NPM | - | - | 933 |  |  | - | - | - | - | - | - |
| FI978322 | 2 | 1 | NPM | *-* | - | 861 |  |  | + | - | - | - | - | - |
| **Hypothetical protein** | |  |  |  |  |  |  |  |  |  |  |  |  |  |
| FI978312 | 1 | 1 | probable secretion protein | *Xoc* BLS256 | 9.0E-29 | 824 |  |  | + | - | - | - | + | + |
| FI978313 | 1 | 1 | hypothetical protein XOO1688 | *Xoo* KACC10331 | 1.0E-14 | 894 | + | + |  | + | + | + | - | - |
| FI978245 | 1 | 2 | hypothetical protein XOO4599 | *Xoo* KACC10331 | 4.0E-07 | 821 |  |  | - | + | + | + | - | + |
| FI978248 | 1 | 2 | hypothetical protein Xoryp_17850 | *Xoc* BLS256 | 2.0E-12 | 464 |  |  | + | + | + | + | + | + |
| FI978249 | 1 | 2 | probable secretion protein | *Xoc* BLS256 | 5.0E-55 | 408 |  |  | + | - | - | - | + | + |
| FI978256 | 1 | 3 | hypothetical protein XAC3725 | *Xac* str. 306 | 3.0E-35 | 846 |  |  | - | - | + | + | - | + |
| FI978269 | 1 | 6 | hypothetical protein XAC3314 | *Xac* str. 306 | 4.0E-54 | 795 |  |  | + | - | - | - | - | + |
| FI978272 | 1 | 5 | hypothetical protein Xoryp_04315 | *Xoc* BLS256 | 3.0E-64 | 409 |  |  | + | + | + | + | + | + |
| FI978278 | 1 | 9 | hypothetical protein XAC0817 | *Xac* str. 306 | 1.0E-39 | 782 |  |  | - | + | + | + | + | + |
| FI978283 | 1 | 17 | hypothetical protein XAC3968 | *Xac* str. 306 | 3.0E-05 | 471 |  |  | - | + | + | + | + | + |
| FI978285 | 1 | 26 | hypothetical protein XOO1934 | *Xoo* KACC10331 | 5.0E-13 | 774 |  |  | - | + | + | + | + | + |
| FI978315 | 1 | 1 | hypothetical protein | *Xoo* KACC10331 | 8.0E-39 | 867 |  |  | - | + | + | - | - | + |
| FI978160 | 1 | 1 | hypothetical protein | *Xoo* MAFF 311018 | 0 | 452 |  |  | - | + | + | + | + | + |
| FI978151 | 1 | 1 | hypothetical protein XOO1934 | *Xoo* KACC10331 | 0 | 366 | - |  | + | + | + | + | + | + |
| FI978079 | 1 | 1 | hypothetical protein XAC0016 | *Xac* str. 306 | 0 | 596 |  | + | + | + | + | + | + | + |
| FI978082 | 1 | 1 | hypothetical protein XAC0817 | *Xac* str. 306 | 0 | 287 | - | + | + | + | + | + | + | + |
| FI978175 | 1 | 1 | hypothetical protein XAC3525 | *Xac* str. 306 | 0 | 292 |  |  | + | - | - | + | + | + |
| FI978063 | 1 | 1 | hypothetical protein | *Xoo* PXO99A | 0 | 1144 | + |  | - | + | + | + | + | + |
| **FI978067** | 1 | 1 | conserved hypothetical protein | *Xoo* MAFF 311018 | 0 | 537 |  | - |  | + | + | + | + | + |
| FI978198 | 1 | 1 | hypothetical protein | *Xoo* MAFF 311018 | 0 | 322 |  | + |  | + | + | + | + | + |
| FI978169 | 1 | 1 | hypothetical protein | *Xoo* PXO99A | 0 | 355 | - |  |  | - | - | + | + | + |
| FI978164 | 1 | 1 | conserved hypothetical protein | *Xoo* MAFF 311018 | 0 | 438 |  | + |  | + | + | + | + | + |
| FI978071 | 1 | 1 | hypothetical protein XOO_3844 | *Xoo* MAFF 311018 | 0 | 507 | - |  |  | + | + | + | + | + |
| FI978304 | 2 | 1 | hypothetical protein XOO1051 | *Xoo* KACC10331 | 1.0E-87 | 823 |  |  | - | + | + | + | + | - |
| **FI978305** | 2 | 1 | hypothetical protein xccb100_3708 | *Xcc* str. ATCC 33913 | 4.0E-18 | 855 |  |  | + | - | - | - | - | + |
| FI978306 | 2 | 1 | conserved hypothetical protein | *Xoo* PXO99A | 2.0E-45 | 805 |  |  | - | - | - | + | - | - |
| FI978307 | 2 | 1 | hypothetical protein XCC2965 | *Xcc* str. ATCC 33913 | 3.0E-12 | 835 | - |  |  | - | - | - | - | - |
| FI978237 | 2 | 2 | hypothetical protein | *Xoo* PXO99A | 8.0E-36 | 843 |  |  | - | + | + | + | + | + |
| FI978251 | 2 | 3 | hypothetical protein XAC1497 | *Xac* str. 306 | 5.0E-07 | 610 |  |  | + | - | - | - | - | + |
| FI978239 | 1 and 2 | 2 | hypothetical protein XCC2966 | *Xcc* str. ATCC 33913 | 7.0E-11 | 244 | + |  |  | - | - | - | - | - |
| FI978240 | 1 and 2 | 2 | hypothetical protein XCC0647 | *Xcc* str. ATCC 33913 | 1.0E-62 | 791 |  |  | + | - | - | - | - | + |
| **FI978252** | 1 and 2 | 2 | hypothetical protein XOO0776 | *Xoo* KACC10331 | 1.0E-57 | 785 |  | + | + | + | + | + | + | + |
| FI978265 | 1 and 2 | 4 | hypothetical protein XCC4108 | *Xcc* str. ATCC 33913 | 3.0E-83 | 500 |  |  | + | **-** | **-** | + | **-** | + |
| FI978289 | 1 and 2 | 34 | hypothetical protein CLOSS21_00494 | *Xoo* PXO99A | 2.0E-20 | 635 | + |  |  | + | + | + | + | + |
| FI978253 | 1 and 2 | 2 | hypothetical protein YintA_01003971 | *Xoo* MAFF 311018 | 3.0E-24 | 802 |  |  |  | + | + | + | + | + |
| **Cell envelope & motility** | |  |  |  |  |  |  |  |  |  |  |  |  |  |
| **FI978319** | 1 | 1 | type IV pilin | *Xoc* BLS256 | 3.0E-13 | 767 |  |  | + | + | + | + | + | + |
| FI978267 | 1 | 5 | fimbrial assembly protein | *Xoo* PXO99A | 1.0E-61 | 638 |  |  | + | + | + | + | + | + |
| FI978162 | 1 | 1 | outer membrane antigen | *Xoo* KACC10331 | 0 | 129 | - |  |  | + | + | + | + | + |
| FI978178 | 1 | 1 | pilin | *Xoo* KACC10331 | 0 | 377 |  |  | + | + | + | + | + | + |
| FI978318 | 2 | 1 | PilY1 protein | *Xoo* MAFF 311018 | 2.0E-31 | 816 |  |  | + | + | + | + | + | + |
| FI978286 | 1 and 2 | 23 | putative glycosyl transferase | *Xoo* KACC10331 | 1E-162 | 1017 |  |  | - | + | + | + | - | + |
| FI978238 | 1 and 2 | 2 | putative glycosyl transferase | *Xoo* KACC10331 | 1.0E-37 | 828 |  |  | - | + | + | + | - | + |
| **Phage related & IS elements** | |  |  |  |  |  |  |  |  |  |  |  |  |  |
| FI978231 | 1 | 2 | ISXo1 transposase (IS5 family) | *Xoo* PXO99A | 7.0E-40 | 839 |  | + | + | - | - | + | + | + |
| FI978232 | 1 | 2 | ISXo1 transposase (IS5 family) | *Xoo* PXO99A | 5.0E-39 | 803 |  | + |  | - | - | + | + | + |
| FI978246 | 1 | 2 | transposase | *Xoo* PXO99A | 2.0E-08 | 937 |  |  | - | + | + | + | + | + |
| FI978241 | 1 | 2 | transposase (IS4 family) | *Xoo* KACC10331 | 8.0E-41 | 825 | - | + |  | + | + | + | + | + |
| FI978254 | 1 | 3 | IS1478 transposase (IS5 family) | *Xoo* KACC10331 | 9.0E-19 | 173 |  | + | - | + | + | + | + | + |
| FI978255 | 1 | 2 | ISXoo5 transposase (IS5 family) | *Xoc* BLS256 | 1.0E-21 | 813 |  |  | + | + | + | + | + | + |
| FI978258 | 1 | 3 | ISXoo15 transposase (IS30 family) | *Xoo* KACC10331 | 6.0E-39 | 746 |  | - |  | + | + | + | + | + |
| FI978261 | 1 | 4 | ISXoo5 transposase (IS5 family) | *Xoc* BLS256 | 6.0E-26 | 340 |  |  | + | + | + | + | + | + |
| FI978262 | 1 | 4 | ISXo8 transposase (IS5 family) | *Xoo* PXO99A | 6.0E-29 | 790 |  |  | + | + | + | + | + | + |
| FI978270 | 1 | 7 | putative transposase | *Xoo* KACC10331 | 1.0E-24 | 488 | + |  |  | + | + | + | + | + |
| FI978271 | 1 | 7 | gene transfer agent (GTA) like protein | *Pl* DS-1 | 8.0E-50 | 788 |  | + |  | - | - | - | - | - |
| FI978274 | 1 | 8 | ISXoo15 transposase (IS30 family) | *Xoo* MAFF 311018 | 4.0E-49 | 695 |  | + |  | + | + | + | + | + |
| FI978276 | 1 | 8 | IS1404 transposase (IS3 family) | *Xoc* BLS256 | 3.0E-27 | 728 |  |  | + | + | + | + | + | + |
| FI978287 | 1 | 26 | IS30 family transposase | *Xoo* KACC10331 | 2.0E-43 | 323 |  |  | + | + | + | + | + | + |
| **FI978288** | 1 | 28 | putative transposase | *Xoc* BLS256 | 1E-164 | 1659 |  | + | + | + | + | + | + | + |
| FI978076 | 1 | 1 | putative transposase | *Xoo* KACC10331 | 0 | 688 | - |  | + | + | + | + | + | + |
| FI978147 | 1 | 1 | ISXoo15 transposase (IS30 family) | *Xoo* MAFF 311018 | 0 | 549 |  | + | + | + | + | + | + | + |
| FI978078 | 1 | 1 | transposase | *Xoo* MAFF 311018 | 0 | 413 |  | + | + | + | + | + | + | + |
| FI978084 | 1 | 1 | putative transposase | *Xoo* MAFF 311018 | 0 | 323 |  | + | + | + | + | + | + | + |
| FI978069 | 1 | 1 | ISXoo15 transposase (IS30 family) | *Xoo* KACC10331 | 0 | 708 | - | + |  | + | + | + | + | + |
| FI978174 | 1 | 1 | putative transposase | *Xoo* KACC10331 | 0 | 144 | - | + |  | + | + | + | + | + |
| FI978106 | 1 | 1 | putative transposase | *Xoo* PXO99A | 1.0E-59 | 281 | - |  |  | + | + | + | + | + |
| FI978083 | 1 | 1 | putative transposase | *Xoo* KACC10331 | 0 | 671 | - |  |  | + | + | + | + | + |
| **FI978099** | 1 | 1 | putative transposase | *Xoo* KACC10331 | 3.0E-23 | 457 |  | + | + | + | + | + | + | + |
| FI978109 | 1 | 1 | IS1478 transposase (IS5 family) | *Xoo* KACC10331 | 0 | 178 | - |  |  | + | + | + | + | + |
| FI978233 | 2 | 1 | ISXo8 transposase (IS5 family) | *Xoo* PXO99A | 2.0E-21 | 887 |  | + |  | + | + | + | + | + |
| FI978250 | 2 | 3 | prophage Lp2 protein 6 | *Xoo* PXO99A | 6.0E-46 | 702 |  |  | - | + | + | + | + | + |
| **Metabolism** |  |  |  |  |  |  |  |  |  |  |  |  |  |  |
| FI978324 | 1 | 1 | haemolysin III | *Xcc* str. ATCC 33913 | 5.0E-17 | 853 | - |  |  | - | - | - | - | - |
| FI978325 | 1 | 1 | secreted xylanase | *Xoc* BLS256 | 9.0E-26 | 707 |  |  | + | + | + | + | + | - |
| FI978242 | 1 | 2 | glycine dehydrogenase | *Xoo* PXO99A | 6.0E-20 | 738 |  | + |  | + | + | + | + | + |
| FI978290 | 1 | 38 | dTDP-glucose 4,6-dehydratase | *Xoo* KACC10331 | 3.0E-69 | 496 |  |  | - | + | + | + | + | + |
| FI978181 | 1 | 1 | cellulase | *Xoo* KACC10331 | 0 | 262 |  |  | + | + | + | + | + | + |
| FI978088 | 1 | 1 | dTDP-glucose 4,6-dehydratase | *Xoo* KACC10331 | 0 | 503 | - |  | - | + | + | + | + | + |
| FI978167 | 1 | 1 | integrase, catalytic region | *Nw* | 0 | 287 | - |  |  | - | - | + | + | + |
| FI978235 | 2 | 2 | putative peptidase, S54 (rhomboid) | *Xoo* PXO99A | 5.0E-33 | 484 |  |  | - | - | - | + | + | + |
| FI978277 | 2 | 10 | ATPase | *Xoo* KACC10331 | 3.0E-45 | 346 |  |  | + | + | + | - | - | - |
| FI978279 | 1 and 2 | 9 | ribonucleoside-diphosphate reductase, beta subunit | *Xoo* PXO99A | 2.0E-29 | 530 |  |  | + | + | + | + | + | + |
| **Signal Transduction** | | |  |  |  |  |  |  |  |  |  |  |  |  |
| FI978266 | 1 | 5 | colicin I receptor | *Xoc* BLS256 | 6.0E-78 | 796 |  |  | - | + | + | + | + | + |
| **Secretion, transport & binding proteins** | | | |  |  |  |  |  |  |  |  |  |  |  |
| FI978326 | 1 | 1 | probable colicin V secretion atp-binding protein | *Xoc* BLS256 | 3.0E-41 | 819 |  |  | - | - | - | - | + | + |
| **FI978328** | 1 | 1 | probable TonB-dependent receptor | *Xoc* BLS256 | 2.0E-49 | 858 |  | + | + | + | + | + | + | + |
| FI978329 | 2 | 1 | TonB-dependent receptor | *Xoc* BLS256 | 8.0E-34 | 858 |  | + |  | + | + | + | + | + |
|  |  |  |  |  |  |  |  |  |  |  |  |  |  |  |
| FI978247 | 1 | 2 | magnesium and cobalt transport protein | *Xoo* KACC10331 | 2.0E-39 | 281 |  |  | - | + | + | + | + | + |
| **FI978259** | 1 | 3 | ATP-binding protein of ABC transporter | *Xoo* MAFF 311018 | 1.0E-32 | 738 |  |  | + | + | + | + | - | + |
| FI978093 | 1 | 1 | probable secretion protein | *Cv* | 0 | 549 |  |  | - | - | - | - | - | + |
| FI978105 | 1 | 1 | probable secretion protein | *Cv* | 0 | 409 |  |  | - | - | - | - | - | + |
| FI978095 | 1 | 1 | ATP-binding protein of ABC transporter | *Xoo* MAFF 311018 | 0 | 520 |  | + |  | + | + | + | + | + |
| FI978102 | 1 | 1 | putative secreted protein | *Xoo* PXO99A | 0 | 293 | - |  |  | + | + | + | + | + |
| FI978243 | 1 | 2 | putative secreted protein | *Xoc* BLS256 | 6.0E-20 | 442 |  |  | - | + | + | + | + | + |
| FI978257 | 1 | 3 | putative secreted protein | *Xcv* str. 85-10 | 3.0E-32 | 231 |  |  | + | + | + | + | + | + |
| FI978275 | 1 and 2 | 9 | colicin V secretion/processing ATP-binding protein CvaB | *Xoo* PXO99A | 8.0E-91 | 637 |  | - | + | + | + | + | + | + |
| **Virulence-related sequence** | | | |  |  |  |  |  |  |  |  |  |  |  |
| **FI978263** | 1 | 3 | HrpF protein | *Xoo* KACC10331 | 3.0E-75 | 744 |  | + | + | + | + | + | + | + |
| **FI978149** | 1 | 1 | Avr/Pth14 (avr/pth14) gene | *Xoc* BLS256 | 9.0E-58 | 887 |  | + | + | - | - | + | + | + |
| FI978260 | 1 | 3 | virulence regulator | *Xoo* PXO99A | 3.0E-47 | 769 |  |  | + | + | + | + | + | + |
| **FI978282** | 1 and 2 | 17 | avirulence protein AvrBs3/pth family | *Xoc* BLS256 | 1.0E-19 | 397 |  |  | + | + | + | + | + | + |
| **ACD57163** | PCR**§** | 1 | XopX effector protein | *Xoo* PXO99A | 0 | 509 | + | + | + | + | + | + | + | + |
| **AF275267** | PCR | 1 | avirulence protein gene (avrXa7) | *Xoo* KACC10331 | 0 | 725 |  | + | + | + | + | + | + | + |
| BAE68417 | PCR | 1 | leucin rich protein | *Xoo* MAFF 311018 | 0 | 390 |  | + | + | + | + | + | + | + |
| ACD60316 | PCR | 1 | Rhs element Vgr protein | *Xoo* PXO99A | 0 | 618 |  | + | + | + | + | + | + | + |

† SSH library and/or libraries in which the clone was identified, where 1 corresponds to SSH library *Xoo* strain M1/PXO86, and 2 to SSH library *Xoo* strain M1/*Xoc* BLS256.

**‡** Number of sequences by contig, where 1 indicates singleton.

†† *Xoo* is *Xanthomonas oryzae* pv. *oryzae*; *Xcc* is *Xanthomonas campestris* pv. *campestris*; *Xoc* is *Xanthomonas oryzae* pv. *oryzicola*; *Xac* is *Xanthomonas axonopodis* pv. *citri*; *Pl* is *Parvibaculum lavamentivorans*; *Nw* is *Nitrobacter winogradsky*; *Cv* is *Chromobacterium violaceum*; *Xcv* is *Xanthomonas campestris* pv. *vesicatoria*.

∥ Time point, in days after inoculation, where + indicates up-regulated, and - indicates down-regulated.

¶*Xanthomonas oryzae* genomes, where + indicates presence of gene homologues to *Xoo* MAI1 in the genome analysed, and - indicates absence.

**§** These DNA fragments were amplified by PCR using primers designed from available sequences in the GenBank database, accessions number are referred in the table.

Clones in bold indicate those that were used for qRT validation.
